# Polycaprolactone Electrospun Nanofiber Membrane with Skin Graft Containing Collagen and Bandage Containing MgO Nanoparticles for Wound Healing Applications

**DOI:** 10.3390/polym15092014

**Published:** 2023-04-24

**Authors:** Sadegh Nikfarjam, Yaqeen Aldubaisi, Vivek Swami, Vinay Swami, Gang Xu, Melville B. Vaughan, Roman F. Wolf, Morshed Khandaker

**Affiliations:** 1 Department of Biology, University of Central Oklahoma, Edmond, OK 73034, USA; 2 School of Engineering, University of Central Oklahoma, Edmond, OK 73034, USA; 3 Oklahoma Veterans Affairs Health Care System, Oklahoma City, OK 73104, USA

**Keywords:** skin graft, polycaprolactone, collagen, bandage, antibacterial, wound healing

## Abstract

The objective of this study was to create a nanofiber-based skin graft with an antimicrobial bandage that could accelerate the healing of an open wound while minimizing infection. To this end, we prepared a bi-layer construct where the top layer acts as bandage, and the bottom layer acts as a dermal equivalent graft. A collagen (CG) gel was combined without and with an electrospun polycaprolactone (PCL) membrane to prepare CG and CG-PCL dermal equivalent constructs. The antibacterial properties of PCL with and without an antibacterial agent (MgO nanoparticles) against *Staphylococcus aureus* (ATCC 6538) was also examined. Human dermal fibroblasts were cultured in each construct to make the dermal equivalent grafts. After culturing, keratinocytes were plated on top of the tissues to allow growth of an epidermis. Rheological and durability tests were conducted on in vitro dermal and skin equivalent cultures, and we found that PCL significantly affects CG-PCL graft biological and mechanical strength (rheology and durability). PCL presence in the dermal equivalent allowed sufficient tension generation to activate fibroblasts and myofibroblasts in the presence of transforming growth factor-beta. During culture of the skin equivalents, optical coherence tomography (OCT) showed layers corresponding to dermal and epidermal compartments in the presence or absence of PCL; this was confirmed after fixed specimens were histologically sectioned and stained. MgO added to PCL showed antibacterial activity against *S. aureus.* In vivo animal studies using a rat skin model showed that a polycaprolactone nanofiber bandage containing a type I collagen skin graft has potential for wound healing applications.

## 1. Introduction

Skin grafting is a frequent treatment for severe skin injuries such as burns, persistent sores, and tissue loss. Traditional skin grafting methods have proven to be effective, but they have drawbacks such as donor site morbidity, graft rejection, and bad cosmetic results. Advances in tissue engineering and biomaterials have resulted in the creation of innovative methods to improve skin grafting results. Nanofiber scaffolds and collagen are potential methods to imitate the natural shape of the extracellular matrix, allowing cells to adhere and proliferate in a supportive environment. Collagen, a protein found in connective tissues, has been shown to improve cell migration and tissue healing. With this information, we might predict that individuals who have suffered severe skin damage could benefit from skin grafting using nanofibers and collagen [1]. Skin equivalents are an in vitro model of the skin used to study wound healing, keratinocyte migration [2], or barrier function [3]. PCL is a synthetic biodegradable polymer used to serve as a scaffold to study the 3D structure of a tissue. During connective tissue growth and repair, the fibroblasts or myofibroblasts monitor and secrete/repair extracellular matrix materials. A PCL scaffold will degrade and allow the formation of functional tissues.

Polycaprolactone (PCL) nanofibers have gained immense attention in tissue engineering as a promising biomaterial due to their biocompatibility, biodegradability, and mechanical properties. PCL nanofibers, when used as skin grafts, have shown excellent wound healing properties, but their effectiveness is limited by their inability to promote cell proliferation and extracellular matrix synthesis. To address this limitation, several studies have focused on enhancing the biological activity of PCL nanofiber skin grafts by incorporating type I collagen. The most prevalent protein in the extracellular matrix of skin tissue is type I collagen and is responsible for maintaining its structure and function. In vitro, the inclusion of type I collagen in PCL nanofiber skin transplants improved cell adhesion, proliferation, and differentiation. In vivo experiments have also yielded encouraging findings for the use of PCL nanofiber skin grafts and type I collagen bandages. Using PCL nanofiber skin grafts containing type I collagen in a rodent model improved wound healing and tissue regrowth. Moreover, it also enhanced wound healing and decreased inflammation in a diabetic rodent model [4,5].

Collagen is a protein composed of amino acids. To create a collagen hydrogel, collagen concentrate, water, concentrated culture media, and cells are combined. This hydrogel is a highly viable option for skin grafts because collagen is the primary structural protein present in the extracellular matrix of the skin. Moreover, extensive research in tissue engineering has been dedicated to collagen owing to its exceptional biological properties. The fabrication process combining a membrane and hydrogel layers was investigated in previous research [5,6]. A skin graft can be fabricated by utilizing multilayers of the hydrogel membrane, fibrous membrane, or a composite scaffold consisting of multiple layers of a fibrous-hydrogel membrane. In this study, we used a bi-layer of a composite skin graft where the top PCL layer acts as bandage, and the bottom CG layer acts as dermal equivalent graft.

PCL is a hydrophobic polymer that lacks the ability to attract or bind cells without functionalization [7]. In contrast, CG is a scleroprotein, which belongs to a family of proteins characterized by low solubility in water. Additionally, CG is enriched with the amino acid glycine, and it is the only protein known to contain a significant proportion of hydroxyproline, which plays diverse roles in cell metabolism and physiology [8]. Thus, we utilized CG in combination with PCL for two primary reasons [9]. Firstly, CG is a biological adhesive material that functions as a sealant for better attachment of cells with PCL nanofibers. Secondly, CG enhances the hydrophobicity of PCL, which can significantly influence the adhesion of cells with PCL NFM [10].

By binding the antimicrobial and osteoinductive molecules to the polycaprolactone (PCL) nanofiber membrane (NFM), prolonged antibacterial and osteoinductive activities of PCL NFM for biomedical applications are made feasible [10]. MgO NP shows promising antimicrobial properties with excellent biocompatibility with osteoblast cells in CG-PCL NFM in our prior study [10]. In this work, the influence of MgO NP-tethered PCL on the antibacterial activity of PCL-NFM was investigated. This study used *Staphylococcus aureus* to compare the antibacterial effects of PCL with and without MgO nanoparticles.

The objective of the study is to assess and enhance the effectiveness of a three-dimensional CG-PCL model, designed to simulate human skin, for evaluating wound therapies. Moreover, this innovative material has potential for utilization in other applications, including intervertebral discs, tendons, and ligaments, with the potential to improve the quality of life for numerous individuals. This study also evaluated the PCL nanofiber alongside MgO for improved wound healing processes. Most importantly, the study conducted a pilot in vivo test using an established rat skin model to test the efficacy of our designed CG-PCL for repairing a skin wound. The future work related to the present is also presented in the discussion.

## 2. Materials and Methods

### 2.1. Materials

PCL beads with a pellet size of approximately 3 mm and an average molecular weight of 80,000 were combined with acetone (a laboratory reagent with a purity of at least 99.5%) to prepare the PCL solution. Both the PCL pellets and acetone were obtained from Sigma Aldrich (Sigma-Aldrich Co., LLC., St. Louis, MO, USA). The CG-PCL construct was made using type I bovine collagen from Gibco. 

Human dermal fibroblasts of lot #00703 and 01035 (vendor’s code for donor to maintain anonymity) and normal human keratinocytes (FC-0064) were purchased from Lifeline Cell Technology, Frederick, MD, USA. DMEM/high glucose (GIBCO) was purchased from Thermo Fisher. FBS was purchased from BioWest, an antibiotic/antimycotic solution and trypsin/EDTA solution from Millipore Sigma. The 10× EMEM was purchased from Lonza Group Ltd., Basel, Switzerland. Collagen (Rat tail, type I) was purchased from Corning chemical. Mouse anti-human alpha-smooth muscle actin (clone 1A4, 1:1000 dilution; Sigma Aldrich) and Goat anti-mouse Alexa 488 (1:200; Thermo Fisher, Waltham, MA, USA) were obtained. The proliferation kit was purchased from BaseClick Gmbh, Munich, Germany. 

### 2.2. Sample Design

Skin equivalents require mechanical tension; thus, we used the PCL nanofiber membrane, suspended within the collagen, to allow tension generation. We prepared skin equivalents in the presence or absence of a PCL-nanofiber membrane. Fibroblasts, collagen, +/− tension ring, (supporter of the skin graft) +/− PCL were mixed together and allowed to polymerize into a dermal equivalent; the culture continued to allow contraction or tension generation. For the in vitro studies, keratinocytes were plated atop the CG and CG-PCL grafts, and a subsequent culture at the air/liquid interface (ALI) was conducted to form CG and CG-PCL skin equivalent samples. For in vivo study, two Wistar rats of the same sex and age were used. One rat received graft-bandage constructs on a 5 mm diameter wound, and the other rat received only the wound. 

### 2.3. Sample Preparation

#### 2.3.1. Nanofiber Membranes

**Figure 1:**
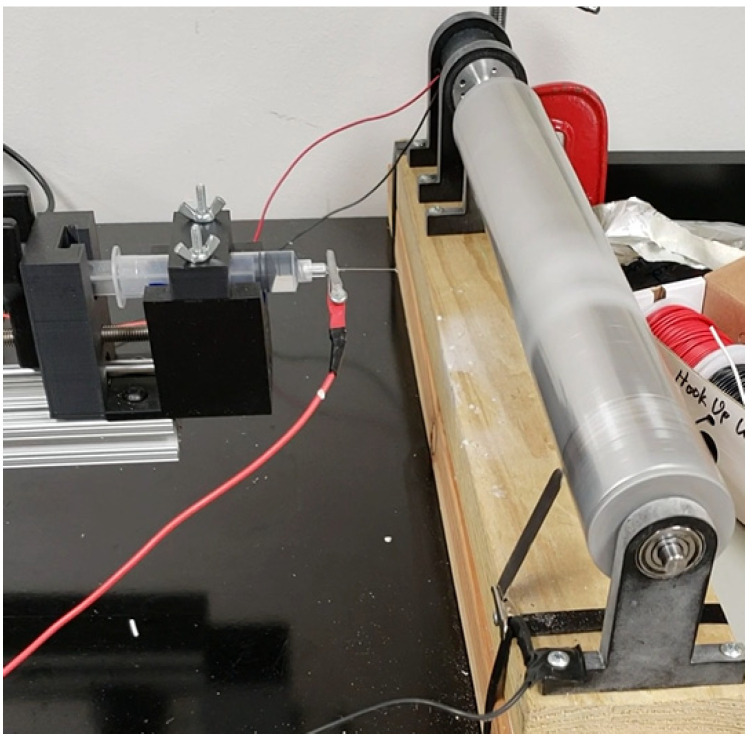
Our custom-made electrospun setup used to produce to the PCL membrane.

PCL and MgO tethered PCL membranes were produced using our custom-made electrospun machine (Figure 1). A PCL solution was used in our custom-made electrospun machine (Figure 1), where 0.5 g of PCL pellets were mixed with 5 mL of acetone solution and sonicated for 30 min for creating the spanning solution. The solution was poured into a custom-made syringe pump with an infuse rate of 0.05 mL/min, and fibers were produced using a 9 kV power supply. The PCL nanofiber cloths were collected on a drum after 5 min of spinning. The cloth was cut into a 10 mm disc using a punch. The 5 wt% MgO nanopowder was sonicated with PCL solutions to make the MgO-PCL solution, which was electrospun to produce MgO-tethered PCL membranes.

#### 2.3.2. Cell Culture

Human dermal fibroblasts were cultured in standard conditions (37 °C in a 5% CO_2_ incubator) in DMEM/high glucose +5% FBS and an antibiotic/antimycotic solution. Normal human keratinocytes were cultured on feeder layers in Rheinwald/Green (RG) media. Fibroblasts and keratinocytes were released from the culture using a 1× trypsin/EDTA solution at room temperature for 5 min. Feeder layers were removed from the keratinocyte culture using pre-warmed 0.02% EDTA/PBS prior to keratinocyte trypsinization. Cells were counted using a standard phase-contrast hemacytometer.

#### 2.3.3. Dermal and Skin Equivalent Model Creation

**Figure 2:**
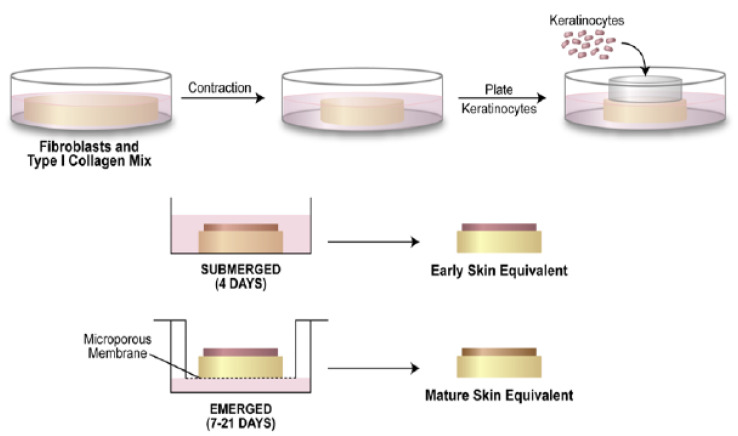
Process of making collagen skin equivalent model [11].

Either HDF 00703 or 01035 were combined on ice with rat-tail type I collagen (Corning), diluted with sterile water, equilibrated with NaOH and 1/10 volume of a concentrated Eagle’s Minimal Essential Medium solution (10× EMEM; Lonza Group LTD) as previously described [11] except we used a 48-well plate and 500 microliters of the mixture plated per well to create the dermal equivalent; final cell concentration was 1 × 10^5^ cells/ml, final collagen concentration was 1 mg/ml (Figure 2). The mixture was plated into pre-warmed wells in the presence or absence of a PCL disc, which was kept submerged using a pipet tip to ensure that collagen was present on both sides of the disc. When the mixture began to become cloudy, the pipet tip was removed, and the plate was placed into the culture incubator to allow continued polymerization for an hour. The dermal equivalents were submerged in growth media and detached from the wells with a pipet tip. Replicate samples were separated to assay for mechanical tension in the tissue, while the remainder were used to build skin equivalents. Following two to four days of submerged culture (until the CG group had contracted about 50%), keratinocytes were added (using a glass cloning cylinder) on top of the tissues to initiate an epidermal layer [11]. Rings were removed after 4 h of keratinocyte attachment to the dermal equivalent upper surface; then, the culture continued to be submerged in RG media for 48 h. The culture was then continued using the air/liquid interface method for up to 3 weeks (in RG media +50 µg/mL ascorbic acid) to mature the tissues fully. The OCT imaging device (Thorlabs, Newton, NJ, USA) used previously to monitor dermal equivalent maturation [12] was modified to monitor the growth of the epidermal layer during culture. After this time, the cultures were fixed in 10% neutral buffered formalin and processed for histological staining.

### 2.4. In Vitro Experiments

#### 2.4.1. Scanning Electron Microscopy (SEM)

Zeiss Neon 40 EsB scanning electron microscopes recorded the fabricated PCL and MgO-PCL membranes’ topographical features. A 5–6 nm layer of AuPd was sputter-coated onto the samples. The SE2 detector was used to collect in-lens pictures while taking SEM photos at 5 kV.

#### 2.4.2. Proliferating Myofibroblast Assay

Proliferation of the myofibroblast in the CG-PCL scaffold was conducted using Doan et al.’s [13] protocol. In short, dermal equivalents were pulsed with 10 µM ethynyl-deoxyuridine (EdU) for 4 h prior to harvesting. Dermal equivalents were then fixed with 4% paraformaldehyde, PBS-washed, and quenched with 0.05M Tris buffer pH 7.4, then permeabilized with ice-cold methanol for 5 min, followed by PBS washes. Samples were blocked with 10% goat serum for 30 min, then stained overnight at 4 degrees C with mouse anti-human alpha-smooth muscle actin followed by PBS washes. Goat anti-mouse Alexa 488 was applied for 45 min, followed by 3% BSA/PBS washes. A Click-stain using Alexa 594 was conducted for 30 min (Base-Click GMBH) followed by PBS washes. Samples were mounted in 80% glycerol/PBS with 14.3 µM DAPI to counterstain the nuclei. Using a DP 72 camera, CelSens software, and an Olympus IX-71 inverted fluorescence microscope, 3-color pictures were captured.

#### 2.4.3. Rheological Tests

Rheological characterization was performed to evaluate the viscoelastic property difference between collagen with no PCL and samples with PCL and collagen. The rheology tests were conducted using a 20 mm diameter cone plate under gap size 1 mm at 25 °C. Before starting each test, the samples were left to reach the equilibrium state on the plate for five minutes to reach the mechanical and temperature equilibrium needed. The test was oscillatory at G’ and G”; the strain sweep amplitude was set at a frequency value of 1 Hz, and the deformation ranged between 0.001 and 10 for 10 min for each sample.

#### 2.4.4. Durability Test

**Figure 3:**
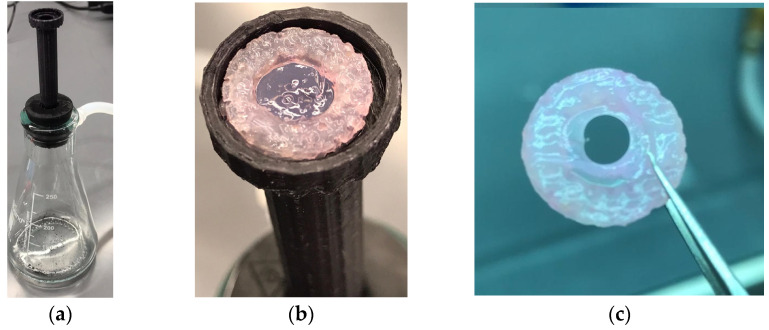
Durability test of a CG graft: (**a**) 3D printed platform to hold the graft for the test, (**b**) sample on the flatform, and (**c**) failed sample after the durability test.

We fabricated a mechanical device that can measure the failure strength of the skin graft under variable pressure and a test of two different skin grafts made with CG and CG-PCL (Figure 3). For testing the durability of each graft, suction was applied to the face of both the CG and CG-PCL. For the suction needed to be applied to the middle of the sample in the same spot for the most accurate results, we designed a 3D-printed platform. The platform had a hollow center so the sample received both suction and gravity applied in the same direction. A suction flask with a plastic stopper was used so that the platform could be inserted at the top with a stopper with a hole at the middle. Tubing was used to attach the vacuum with a regulator and suction gage mounted to the regulator. The other side was attached with similar tubing and connected the suction flask to the regulator. The regulator allowed suction to increase slowly to measure the point at which samples failed accurately. The suction test compared the durability of the CG and CG-PCL grafts.

#### 2.4.5. Antimicrobial Properties

Agar disc diffusion, microbial penetration, and growth kinetic methods were used to determine the antibacterial properties of PCL and MgO-tethered PCL (MgO-PCL). The agar disc diffusion method entailed reducing a bacterium suspension to 10^8^ CFU/mL and distributing it onto an infusion agar dish. Circular samples were sterilized and carefully put on the inoculated plates, and the diameters of the inhibition zones around the discs were measured using a Vernier caliper after 24 h of incubation at 37 °C. The procedure was carried out three times. In the microbial penetration test, an autoclaved nutrient broth, test containers, cotton balls, and transmittance at (600 nm) were used. To assess the film’s ability to prevent microbial penetration, each was firmly put on an open test tube holding a 5 mL autoclaved nutritional broth (NB) medium. To conduct an experiment, two test tubes were utilized for control. The first test tube had no lid or covering and contained NB, which served as the positive control. The second test tube, which also contained NB, was tightly sealed with a cotton ball to serve as the negative control as well as the test groups labeled +PCL/MgO-PCL and −PCL/MgO-PCL based on the test condition (closed environment vs. open environment). The tubes were kept in an open environment for 1 week. The absorbance of NB was determined at 600 nm as a sign of microbial contamination. Nutrient liquid, a bioreactor, a spectrometer, and nano fibers were used in the growth kinetics test. The *S. aureus* suspension was adjusted to a final concentration (10^5^–10^6^ CFU/mL) in nutrient broth. The optical density (OD) at λ = 600 nm was monitored at regular periods (0, 1, 2, 4, and 24 h) using a UV-Vis spectrophotometer to determine the development of *S. aureus* in broth media. The test was performed 10 times and reflected as series 1–10.

### 2.5. In Vivo Animal Studies

#### 2.5.1. Animal Study

The primary objectives for this pilot in vivo animal study were twofold: first, to evaluate in a culture dish (in vitro) the effectiveness of PCL being incorporated into a graft tissue composed of CG and Wistar rat fibroblasts; and second, to investigate how well the CG-PCL tissue works as a bandage and graft for Wistar rat skin. The Institutional Animal Care and Use Committee (IACUC) of the Oklahoma City Department of Veterans Affairs gave its approval to the animal protocol. Adult inbred Wistar rats, both female, young adult, between 200 and 350 g, were purchased from a commercial vendor. Two rats were used for this study, named as the control and test animals. The control rat received only the wound, whereas the test rat received the rat fibroblast seeded CG graft and PCL bandage on the created wound. For the test rat, a CG skin equivalent model was created on 5 mm diameter and 0.5 micrometer thickness Polycaprolactone (PCL) nanofiber matrices made by an electrospinning process. The fibroblasts were extracted from our purchased Wistar rat from a separate study using a standard cell extraction protocol and plated within a CG-PCL graft for 14 days. After anesthesia, the rat backs from two rats (control and test) were shaved and the skin disinfected; then, with a biopsy punch, two identical 5-mm full-thickness cutaneous wounds were made on the dorsal skin. For the test rat, the wound was covered by a CG-PCL construct, and the constructs were secured using 3M biological glue. The wound area images were captured after 1, 3, 5, 12, and 28 days. Both control and test rats were euthanized with CO_2_ after 28 days. The extracted skin was dissected into small pieces for histological analysis. The area of the skin graft and the surrounding tissues were collected and analyzed with histopathologic techniques.

#### 2.5.2. Wound Healing Measurement

ImageJ software measured the wound area from the captured images on day 0 (after surgery), 1, 3, 5, 12, and 28 days (before euthanasia). The percentage of wound contraction was measured using the equation: (A_i_ − A_0_) × 100/A_0_, where A_0_ is the day 0 wound area, and A_i_ is the wound area in day i.

#### 2.5.3. Histological Analysis

After fixation, specimens were paraffin-embedded, and serial sections of 4–5 μm were cut using a Leica 2030 manual microtome and mounted on a slide followed by deparaffinization and rehydration of slides to distilled water. After 1 min of Mayers Hematoxylin staining, the slides were rinsed with 4–5 changes of tap water. Slides were then decolorized with three dips in 0.5% acid alcohol followed by washing in 3 changes of distilled water. For 1 min, slides were dipped into the bluing reagent and followed by 3 changes of distilled water. Next, it was placed in eosin which is a fluorescent dye derived from fluorescein and transferred into a solution of 95% ethyl alcohol for 2 min twice. Similarly, 100% ethyl alcohol was used twice for 1 min each. Once this was complete, the slide was finally mixed into 100% xylene. The histologic slide was officially left to dry and mounted with Permount and a coverslip. This process was continued for all the sectioned microscopic slides every 5 min. For morphological observation and morphometric examination of the epidermis and dermis thickness, hematoxylin and eosin (H&E) staining was utilized.

### 2.6. Statistical Analysis

Microsoft Excel statistical tools were used to run independent sample *t*-tests, assuming unequal variances, to check for variations in mean adhesion density, cell proliferation, differentiation, and degradation among the sample groups. All tests had a significance threshold of 0.05.

## 3. Results

### 3.1. In Vitro Studies

#### 3.1.1. SEM Images

**Figure 4:**
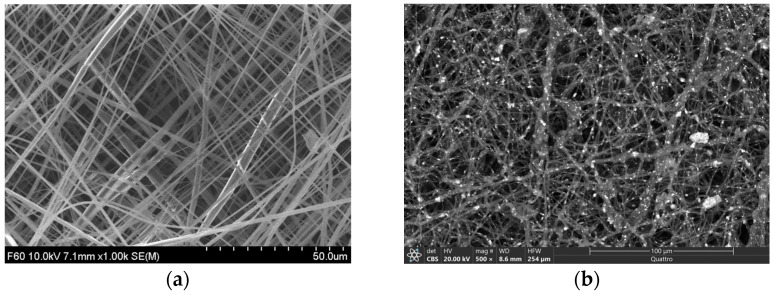
SEM images of a PCL (**a**) and PCL with MgO (**b**).

To analyze internal morphology, in particular the attachment of MgO with PCL fibers, scanning electron microscopy (SEM) images of the PCL top surface were taken without (Figure 4a) and with MgO nanoparticles (Figure 4b). The SEM image of the PCL membrane without and with MgO showed porosity and random fiber orientation. The nanofiber diameter was measured using ImageJ to about 1.9–3.2 µm.

#### 3.1.2. PCL Allows Tension Generation in Dermal Equivalents

**Figure 5:**
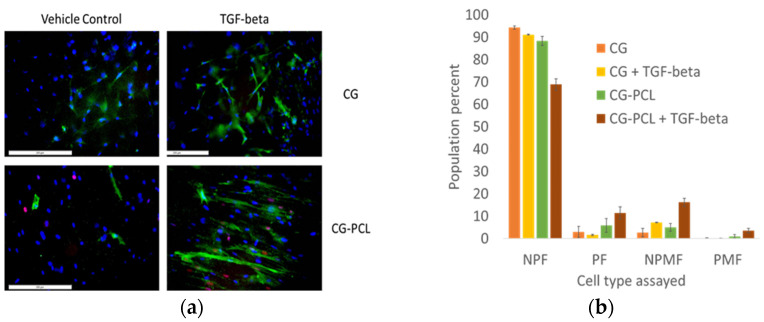

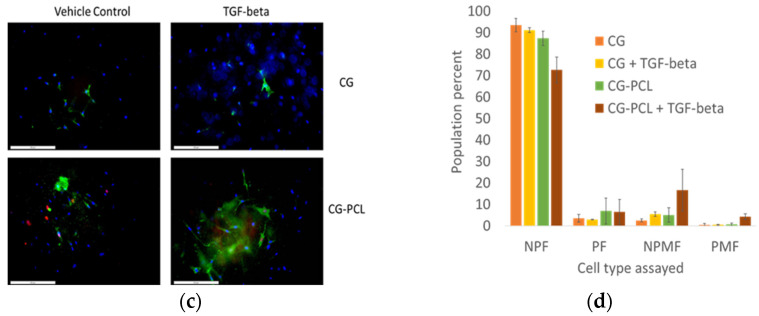
HDF00703 (**a**,**b**) and HDF01035 (**c**,**d**) proliferation and myofibroblast differentiation assay for CG and PCL-CG in the presence or absence of transforming growth factor-beta. Left images show the myofibroblast stain result; right graphs show the quantified cell type per treatment. Note the increased presence of activated and differentiated myofibroblasts when CG-PCL was treated with TGF-beta. NPF = non-proliferating fibroblast; PF = proliferating fibroblast; NPMF = non-proliferating myofibroblast; PMF = proliferating myofibroblast. Scale bar = 200 µm.

CG dermal equivalents had contracted to 50% of their original size; however, the diameter of CG-PCL was similar to the diameter of the PCL itself; this indicated that the cells were unable to contract the PCL, allowing tension to generate. This was evident in stained CG-PCL whole-mounts which showed the presence of well-spread fibroblasts having stress fibers present [14], unlike the CG alone. A total of four cell types were identified with the combined alpha-smooth muscle actin and EdU-Alexa 594 stain (Figure 5a,c): nonproliferative fibroblasts (NPF; blue nucleus only), proliferative fibroblasts (PF; pink nucleus only), nonproliferative myofibroblasts (NPMF; green cytoplasmic fibers and blue nucleus), and proliferative myofibroblasts (PMF; green cytoplasmic fibers and pink nucleus). All images were quantified (Figure 5b,d). In all treatments, most cells were NPF; however, upon stimulation with TGF-beta, NPMF slightly increased in CG, while all activated cell types (PF, NPMF, PMF) increased in CG-PCL. 

**Figure 6:**
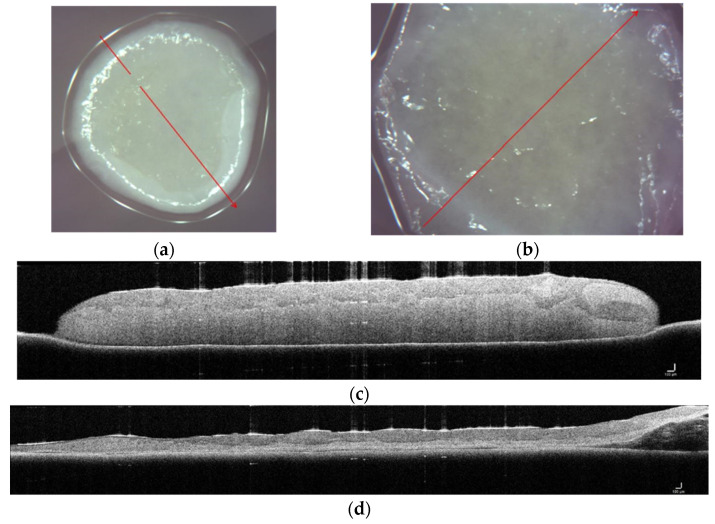
Z-axis image showing dermal and epidermal layers in cultured skin equivalents. CG skin equivalents (**a**,**c**) demonstrated thicker, smaller-diameter tissues (c, lower light layer) with a stratified epithelium atop (c, higher, dark layer). CG-PCL (**b**,**d**) showed a similar pattern although the entire tissue was thinner with a larger diameter. The length of scale bar is 1 µm in image (**c**,**d**).

Skin equivalents were viewed using OCT while still in culture to determine if the technique would be an informative predictor of tissue organization similar to its use with human skin [15,16]. Overhead images of CG (Figure 6a) and CG-PCL (Figure 6b) were used to direct the placement of a red line where the z-axis cross-section was to be developed. These cross-sections of CG (Figure 6c) and CG-PCL (Figure 6d) showed the presence of layers, a dark upper layer, and a bright lower layer. The layering was characteristic of dermal and epidermal compartments as demonstrated by histological sections [3,17]. 

**Figure 7:**
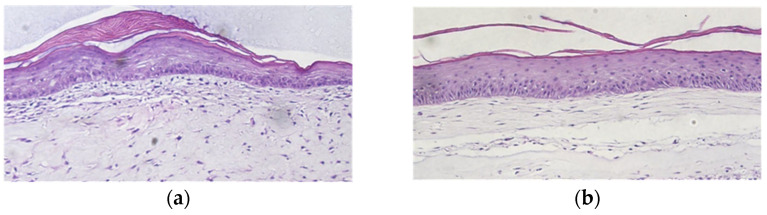
PCL allowed epidermal formation in skin equivalents. H&E-stained cross sections of CG skin equivalents (**a**) demonstrated a stratified squamous keratinized epithelium. Similar strata were produced in PCL-CG skin equivalents (**b**). Note the alignment of elongate fibroblast nuclei in the CG-PCL suggesting tissue tension along a linear plane was present. 100× total magnification.

Next, we fixed, processed, sectioned, and stained cross-sections of the OCT-imaged skin equivalents to determine how PCL affected skin equivalent organization. CG cross-sections showed a thick dermal compartment with a stratified squamous keratinized epithelium (Figure 7a); this organization compared favorably to the OCT cross-sections. CG-PCL showed a similar pattern as CG, although the depth of the dermis was diminished.

#### 3.1.3. Mechanical Properties of CG and CG-PCL

**Figure 8:**
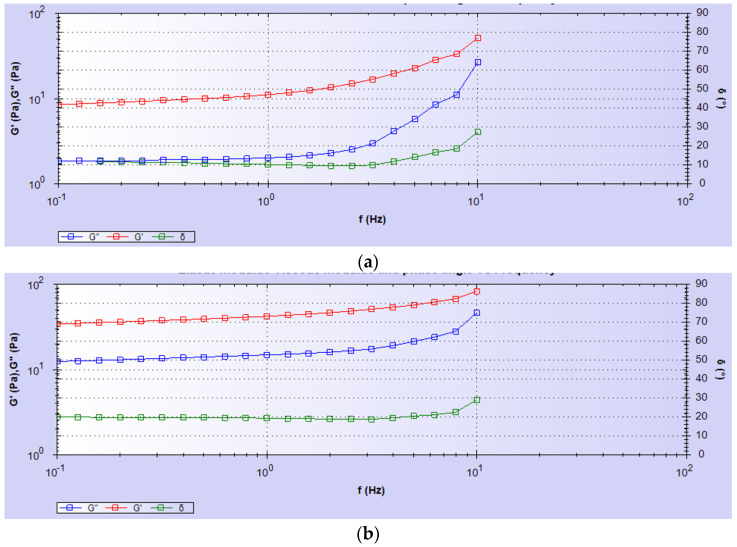
Elastic modulus, viscous modulus, and phase angle vs. frequency of (**a**) CG and (**b**) CG-PCL samples.

We observed differences in rheological properties between CG and CG-PCL samples (Figure 5) for a range of frequencies (0.1 Hz to 10 Hz) as shown in Figure 8. Table 1 shows the difference in values of the shear modulus (G’), complex modulus (G*), and phase angle (δ) of CG graft compared to CG-PCL. We found statistically significant differences in the shear modulus (G’), complex modulus (G*), and phase angle (δ) of CG samples compared to CG-PCL (*p* < 0.05, n = 3).

**Figure 9:**
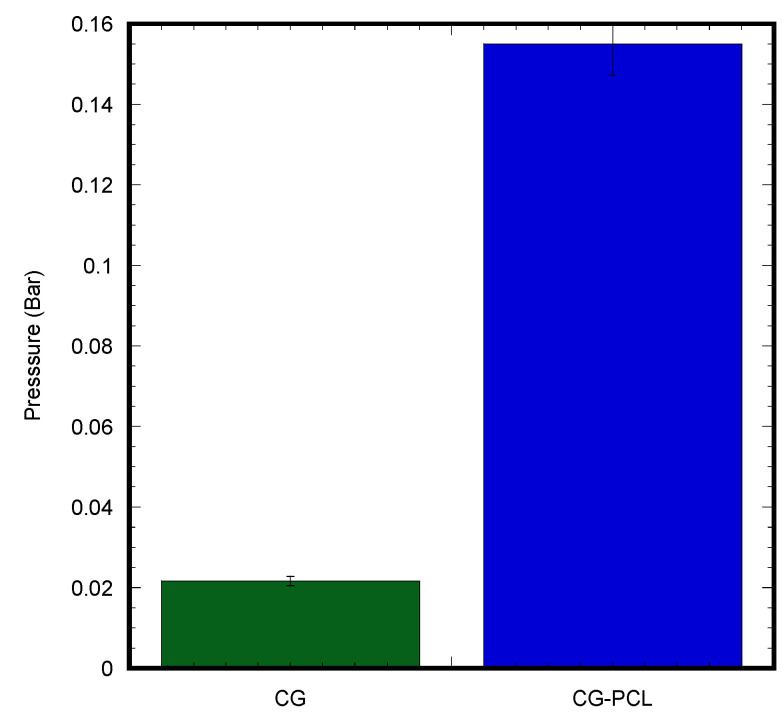
Durability test results of CG and CG-PCL samples.

A statistically significantly higher mechanical strength of CG-PCL was found compared to CG from the durability tests (Figure 9). CG-PCL could withstand more than seven times the suction pressure compared to CG.

The above mechanical tests confirmed that PCL increased the mechanical strength of the CG gel.

#### 3.1.4. Antibacterial Analysis 

**Figure 10:**
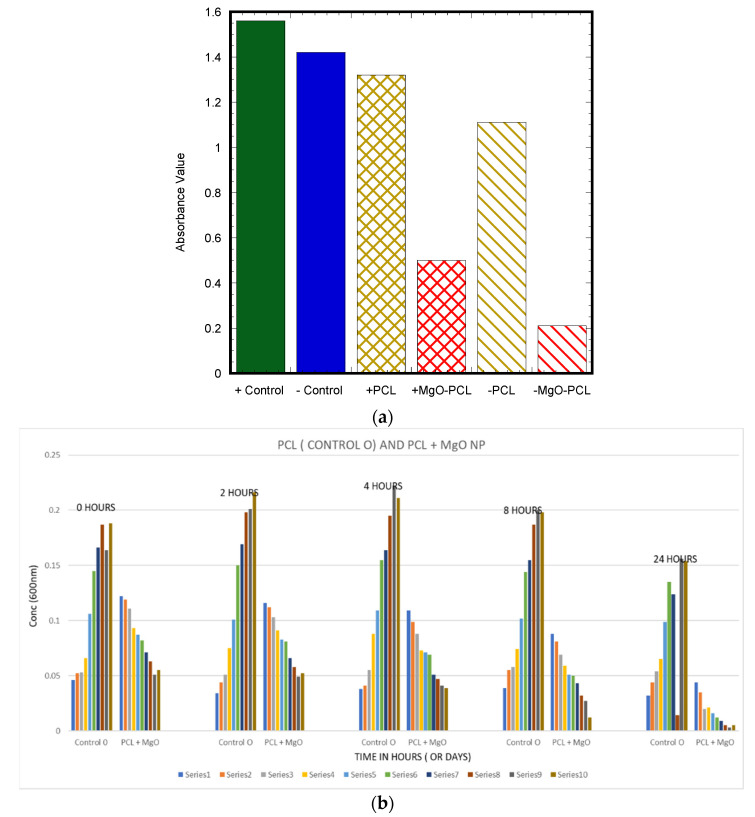
(**a**) Quantification of the Microbial Penetration test, indicating that the microbial activity rate decreased over time for both the negative and positive conditions. (**b**) Growth kinetics results showed growth of bacteria increased in control (PCL), where the bacterial growth decreased with time (1–10 series) for MgO-tethered PCL samples.

The antibacterial activity was estimated using a disk diffusion test, microbial penetration test, and growth kinetic models. The agar disc diffusion test demonstrated no inhibition zones for only PCL samples, whereas MgO-tethered PCL showed a 0.9 mm inhibition zone in bacteria cultured overnight in tryptone soya agar (TSA) media. The PCL MgO nanofiber membrane exhibited higher antibacterial activities against *S. aureus* compared to the PCL nanofiber membrane (Figure 10a). Most importantly, the antibacterial activity was improved with time (Figure 10b). 

### 3.2. In Vivo Studies

**Figure 11:**
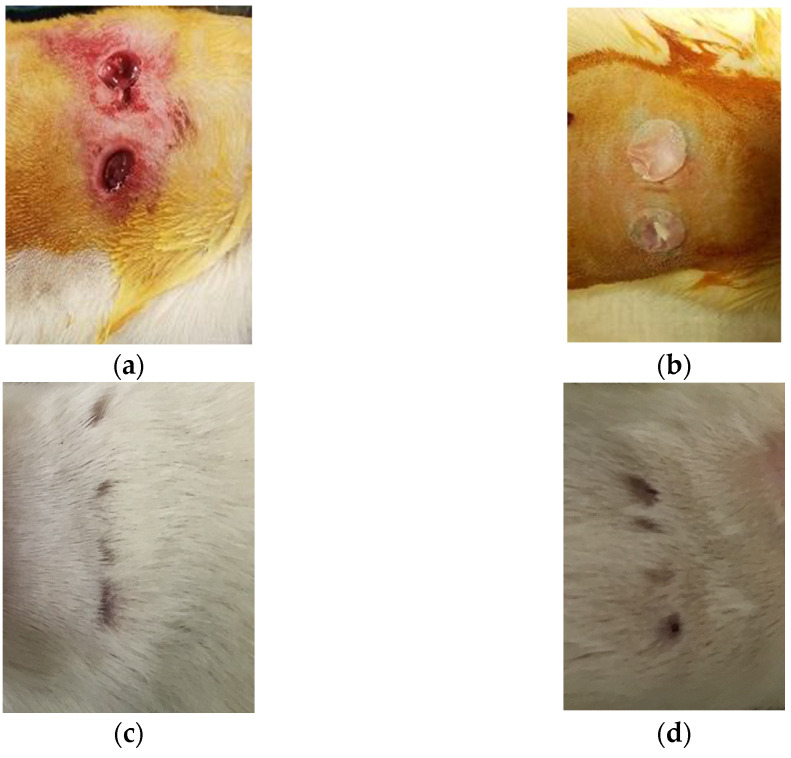
Rats after surgery: (**a**) control and (**b**) test rat. Rats after 28 days of surgery: (**c**) control and (**d**) test rat.

In this investigation, the control and test rats showed no postoperative recovery complications and remained in good condition (Figure 11). The skin of the control and test animals had no visible indications of inflammatory or harmful tissue responses when they were retrieved. Image analysis measured the percentage of the wound healing contraction area with respect to the surgery wound area for the control and test animal. Table 2 reported that the percentage of the contraction area was found to be higher for the CG-PCL grafted rat (test sample) compared to the wound without the graft (control).

**Figure 12:**
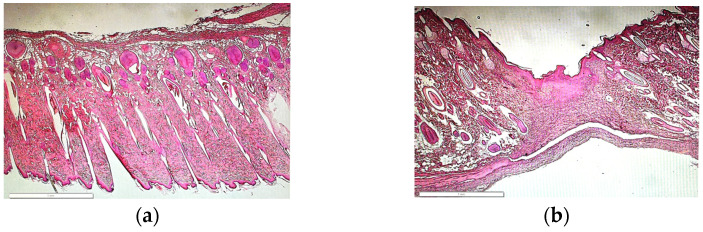
In vivo H&E histology (**a**) control and (**b**) test animal skin. Both images were taken at 4× magnification.

Between fragments from the control and test samples, there were no discernible morphological variations. The samples showed clearly defined borders between the dermis and epidermis (Figure 12). However, some variations in dermal and epidermal thickness were detected. According to ImageJ, the native sample had a larger epidermis and dermis compared to the test sample.

## 4. Discussion

Our goal was to show that PCL could promote a functional tissue beginning with in vitro testing. First, we showed that cells in the presence or absence of PCL could reorganize collagen through compliant matrix contraction. This is an active process of fibroblasts using tractional force migration [18]. While CG continued to contract uninhibited, CG-PCL stopped when contraction reached the PCL diameter, as reported earlier [19,20]. PCL added to CG increases the tensile strength of a dermal equivalent [19]. These observations indicated that mechanical tension was being generated in our CG-PCL constructs. Previous work shows that fibroblasts can differentiate into myofibroblasts in the presence of an anchored matrix [21]. Conversely, the presence of proliferating myofibroblasts indicates the presence of mechanical tension in the tissue [22,23,24]. In the presence of TGFß, NPMF increased in CG; however, all activated phenotypes (PF, NPMF, and PMF) increased in CG-PCL. Previous work showed that proliferation increases in a dermal equivalent under tension [25]. This evidence suggested that there was sufficient mechanical tension necessary to provide the permissive environment for fibroblast and myofibroblast activation [25,26]. The matrix itself was not fibrotic until an external stimulus was applied (TGF-beta); this was also demonstrated in an in vitro model of pulmonary fibrosis where a fibrotic exudate added to nanofibers was required to induce myofibroblasts [27]. Under similar constraints, human mesenchymal cells differentiated into myofibroblasts [28]. PCL scaffolds were also shown to induce myofibroblasts from heart valvular interstitial cells [29]. 

Next, we demonstrated that both CG and CG-PCL could promote maturation of a skin equivalent. Both sample types demonstrated a stratified squamous keratinized epithelium. This was shown in an earlier paper using a CG-PCL pre-polymerization mixture [19] and combined with partial bioprinting [20]; interestingly, viability decreased when PCL became the major component of the mixture. We used OCT to monitor epidermal growth while skin equivalents were culturing. The samples showed a similar z-axis contour as a histologically prepared cross-section. OCT use to study epidermal morphogenesis in vitro during culture was only recently published [30,31] and agrees with our results. It is notable that CG-PCL dermal fibroblast nuclei were aligned and elongated, suggesting the presence of tissue tension parallel to the long axis of nuclei organization, as established in a recent publication [32]. CG dermal fibroblast nuclei, on the other hand, showed no such organization, instead having rounder nuclei and random orientation, suggesting random organization. These results compare favorably to a recent study [32] showing the beneficial effect of tension maintenance to skin equivalent morphogenesis when compared to compliant culture. Future studies with skin equivalents +/− PCL will allow growth factor signaling studies in the presence or absence of tension [33] using this simple and inexpensive modification. Recent evidence demonstrated that human fibroblasts plated directly onto PCL inhibited activation [34]. Proliferation, myofibroblast differentiation, even cell shape were changed compared to cells plated on tissue culture plastic. The contrasting evidence we present is likely due to the cells interacting with the collagen; we were unable to determine if cells attached to the PCL were among those we quantified. It is clear the phenotype demonstrated using immunofluorescence matches the elongate fibroblast interacting with dermal collagen in the histology images. It would be interesting to determine if different integrin subunits engage PCL than collagen or whether other membrane signal transduction molecules might interact with PCL. 

The effectiveness of the antibacterial properties increased as the concentration of nanoparticles increased. This study discovered a viable solution for producing and eliminating bacteria by impregnating the PCL nanofiber membrane with MgO nanoparticles. The most significant finding was that the PCL MgO nanofiber membrane demonstrated notably stronger antibacterial effects against *S. aureus* than the PCL nanofiber membrane. 

In vivo studies demonstrated the effectiveness of polycaprolactone (PCL) nanofiber skin grafts and bandages containing type I collagen in promoting wound healing and tissue regeneration. The present study contributes to the growing body of evidence supporting the potential clinical application of these materials. 

Despite the hopeful findings of these studies, both in vitro and in vivo, there are certain limitations to using PCL nanofiber skin transplants and type I collagen bandages. These materials’ long-term biocompatibility and biodegradation behavior need to be studied further to evaluate their safety and effectiveness for clinical use. The current research adds to the proof that PCL nanofiber skin grafts and bandages containing type I collagen may have a therapeutic utility in skin tissue regeneration research [35,36,37,38,39]. These materials have the potential to be used in a variety of wound healing situations, including those requiring nerve regeneration. More study is needed to optimize these materials for clinical use as well as to evaluate their long-term safety and effectiveness. Future studies are also needed to evaluate swelling, deswelling, and porosity properties of the skin equivalent and their effects on cells. 

Non-healing wounds that persist over long periods represent a significant and escalating burden for healthcare systems worldwide. Consequently, there is an urgent need for innovative therapies that address this issue. To this end, we are currently investigating a novel three-dimensional skin equivalent model called CG-PCL. Utilizing CG-PCL as a skin equivalent model will not only advance our understanding of human skin repair and regeneration but will also contribute towards the development of a new skin graft material for treating damaged skin. The CG-PCL model holds promise for further refinement as the PCL and collagen components can serve as a substrate for growth factors such as fibronectin and anti-bacterial nanoparticles such as magnesium. This enhancement may pave the way for the development and preclinical evaluation of CG-PCL as a viable wound therapy.

## 5. Conclusions

We observed that CG dermal equivalents attached with the PCL nanofiber membrane were able to generate tension similar to tissues with a CG attached in a plastic ring. The epidermis of skin equivalents generated from the bi-layer CG-PCL graft appeared better than CG. The mechanical strength of our developed CG-PCL graft was significantly higher compared to the CG graft. The study found a potential advantage of adding antimicrobial agents with PCL, such as MgO, to improve PCL antibacterial properties. The animal study indicates that the bi-layer CG-PCL graft can be applied in vivo. The results suggest that using the appropriate MgO (antibacterial agent) concentration with the PCL membrane might effectively suppress bacterial growth, as demonstrated by the in vitro antibacterial study. In the future, in vivo studies using antibacterial agents included with the CG-PCL graft will be investigated for designing a unique skin graft with an antibacterial bandage. Evaluation of the unique CG-PCL as a skin equivalent model will not only advance the current understanding of human skin repair and regeneration, but this study will also lead to a new skin graft material for damaged skin in the future project. In particular, further improvement of the CG-PCL model is possible since the PCL and collagen can serve as a place for growth factors (e.g., TGF-beta, fibronectin) and anti-bacterial nanoparticles (e.g., gentamicin, silver) that can aid development and preclinical evaluation of the CG-PCL model for wound therapies.

## Figures and Tables

**Table 1:** Rheological properties’ differences between G and CG-PCL samples at 1 Hz frequency.

Rheological Properties	CG	CG-PCL
Shear modulus (G^’^), Pa	8.64 ± 1.50	37.61 ± 0.87
Viscous modulus (G*), Pa	13.76 ± 2.39	59.87 ± 1.39
Phase angle (δ), Degree	11.99 ± 0.37	18.07 ± 1.60

**Table 2:** Measured percentage of wound healing contraction area with respect to surgery wound area for the control and test animal.

Days	Control		Test	
	Wound # 1	Wound # 2	Wound # 1	Wound # 2
1	10.71	9.91	14.53	11.50
3	34.78	38.67	55.25	35.62
5	46.50	48.28	74.51	78.54
12	62.59	70.63	82.50	82.49
28	80.17	83.19	90.83	91.16

## Data Availability

Data presented in this study are available upon request.
